# Reappraisal of the Dilution and Amplification Effect Framework: A Case Study in Lyme Disease

**DOI:** 10.1002/ece3.71969

**Published:** 2025-08-12

**Authors:** Shirley Chen, S. Eryn McFarlane

**Affiliations:** ^1^ Department of Biology York University Toronto Canada

**Keywords:** amplification effect, biodiversity–disease, dilution effect, disease ecology, hypothesis testing, latent variables, Lyme disease, philosophy of science

## Abstract

The role of biodiversity in regulating zoonotic disease in ecological communities has been broadly referred to as the biodiversity–disease relationship in disease ecology. Whether biodiversity decreases or increases disease risk, known as a dilution or amplification effect respectively, remains unclear. The literature has focused on the strength, generality, nature, and context dependencies that could explain contradictory evidence. We suggest that a continued focus on this approach to resolving the biodiversity–disease debate detracts from a more foundational problem with testing these dilution and amplification hypotheses, in that these hypotheses are not falsifiable as proposed. When tested and interpreted as net effects in a system, these hypotheses do not possess a true null outcome; they are vulnerable to *ad hoc* explanations. Specifically, that an empirical null outcome can be explained by multiple processes (i.e., a true null vs. a canceling out of amplification and dilution effects) means that process cannot be inferred from pattern. To remedy this problem, we propose that biodiversity and disease risk can be modeled as latent variables in multivariate causal models to reframe how we understand them and test the relationship between them. We present a case study on Lyme disease (LD) through a systematic review, concluding that testing these net effect hypotheses falls short of providing robust evidence for its underlying mechanisms. While these hypotheses have previously been helpful in conceptualizing this idea of biodiversity as a potentially protective factor for human health, they require further specificity moving forward in order to appropriately test the relationship.

## Graphical abstract

The biodiversity–disease relationship posits two hypotheses: the dilution effect (where there's a negative relationship between biodiversity and disease risk) and the amplification effect (where there's a positive relationship). However, the literature has historically treated these hypotheses as either/or, ignoring the potential for a null result. We suggest that this is a philosophical fallacy, as all hypotheses must be falsifiable, and suggest strategies to continue to test the biodiversity–disease relationship in wild studies.

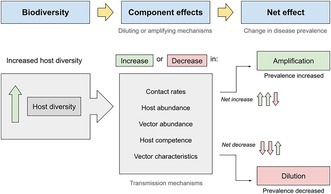

## 1 The Biodiversity–Disease Debate

Wildlife populations are responsible for the emergence of over half of the infectious disease events that threaten human health globally (Jones et al. 2008). Links between anthropogenic activity and biodiversity loss have led disease ecologists to converge on one fundamental principle in the field: changing ecological communities affect the transmission of zoonotic (i.e., non‐human animal originating) diseases (Allen et al. 2017; Estrada‐Peña et al. 2014; Glidden et al. 2021; Keesing et al. 2010; White and Razgour 2020). Following this theoretical basis, two hypotheses have been proposed to suggest that biodiversity loss can either increase or decrease infectious disease risk through what are known as dilution or amplification effects, respectively (Johnson et al. 2015; Keesing et al. 2006; Keesing and Ostfeld 2021; Ostfeld and Keesing 2000b, 2000a; Schmidt and Ostfeld 2001).

While these broad biodiversity–disease relationships (hereafter “diversity‐disease relationships”) have existed before their applications in zoonotic disease systems (Wolfe 1985), criticisms have emerged about the simplicity of the dilution and amplification hypotheses in failing to capture the complex mechanisms and dynamic community interactions within these systems. When proposed as net effects on a system, the ‘dilution effect hypothesis’ suggests that an increase in host diversity limits overall pathogen transmission in ecological communities, whereas the ‘amplification effect hypothesis’ suggests that it would increase overall transmission (Box 1; Keesing et al. 2006). The contradictory nature of these net effect hypotheses has also prompted debates surrounding the context dependencies, generality, and underlying principles of the dilution effect (Civitello et al. 2015; Halliday et al. 2020; Halsey 2018; Ostfeld 2013; Ostfeld and Keesing 2013; Randolph and Dobson 2012; Salkeld et al. 2013; Strauss et al. 2015; Wood et al. 2017; Wood and Lafferty 2013). In attempts to remediate disagreement in the literature, research recommendations abound (Halliday et al. 2020; Johnson et al. 2015; Keesing and Ostfeld 2021; Kilpatrick, Salkeld, et al. 2017; Rohr et al. 2020; Stewart Merrill and Johnson 2020), but ultimately affirm the notion that dilution and amplification hypotheses are inherently testable when proposed as net effects in their current framework.

**FIGURE 1:**
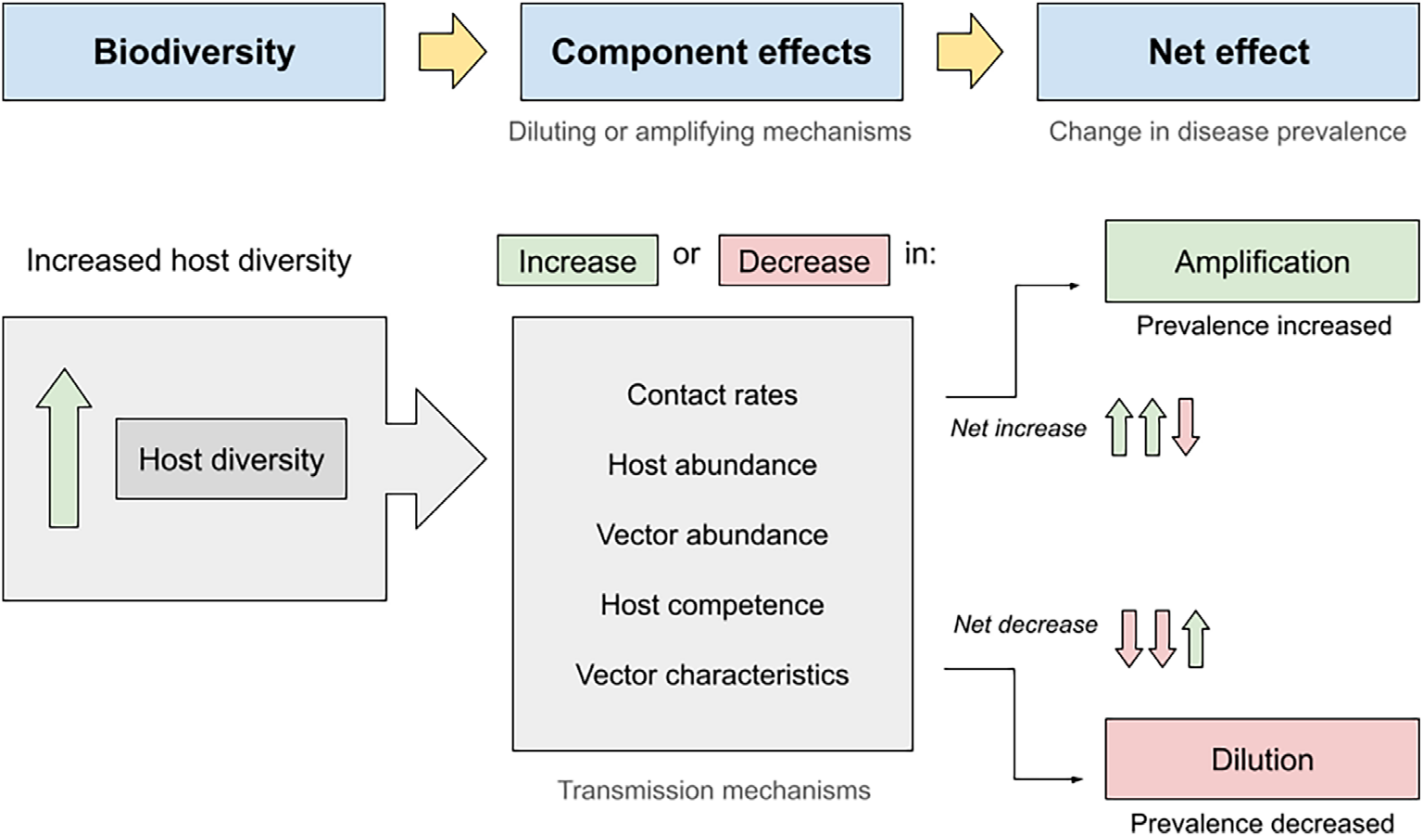
A change in biodiversity, represented specifically by host diversity, can affect the strength of component effects that can increase (amplify) or decrease (dilute) disease transmission. These component effects represent mechanisms operating at a community level and have variable influence on disease transmission. The relative strength of specific mechanisms depends on whether frequency‐dependent or density‐dependent transmission characterizes the system (Rudolf and Antonovics 2005). In frequency‐dependent transmission, host competence and vector characteristics are more important for promoting transmission, independent of their overall population abundances (i.e., population density). On the other hand, density‐dependent systems are more impacted by the population abundances of hosts and vectors to promote transmission through increased contact rates (Dobson 2004; Ostfeld and Keesing 2012). The outcome of all component effects on transmission occurring simultaneously in a system will produce a net increase or decrease in transmission to affect disease prevalence (i.e., disease proportion).

BOX 1 Dilution and amplification effects represent two net outcomes of the same framework describing the relationship between biodiversity and disease risk (Figure 1). In other words, a dilution effect represents a net decrease in disease risk with increasing biodiversity, whereas an amplification effect represents a net increase. These reciprocal net effects result from the cumulation of multiple component effects in a system to primarily mediate the proportion of competent hosts present to support disease transmission. As a result, a change in average community competence is hypothesized to drive a net increase or decrease in disease transmission (Keesing et al. 2006; Ostfeld and Keesing 2000b).

## 2 The Dilution Versus Amplification Debate Cannot Be Resolved as Proposed

Conceptual frameworks describing the dilution and amplification effects have been used to visualize how various mechanistic pathways contribute to an overall net change in disease transmission in the community (Box 1; Keesing et al. 2006; Kocher et al. 2023; Luis et al. 2018; Wood and Lafferty 2013).

These conceptual frameworks have also been useful to help outline the necessary prerequisites for a dilution effect, in particular. Ostfeld and Keesing (2000b) proposed that the following criteria must be met in vector‐borne disease systems in order for a dilution effect to be considered: (1) the vector is a generalist such that host selection is random and host diversity correlates positively with more feeding opportunities, (2) pathogen transmission is horizontal such that transmission occurs from vector blood meals only and not between parent‐offspring generations, (3) host competence varies in the community such that hosts differ in their likelihood of transmitting the pathogen, and (4) there is a positive correlation between host competence and proportional abundance such that the most competent host is proportionally less abundant when species richness increases in the community. Additional prerequisites have been further supplemented into the literature to specify that diverse communities provide species that reduce the maintenance and transmission capability of amplifying hosts, vectors, or the pathogen itself (Johnson et al. 2015). These prerequisites imply that the driving force for the dilution effect is a change in average community competence with a change in host diversity. Conceptually, this reasoning is logical and general enough that this broader mechanism can be attributed to multiple different, individual causal effects of increased host diversity. However, there is also a tendency toward tautology in this reasoning to suggest that this phenomenon occurs only when the conditions in the system allow it to occur. This is not to suggest that many zoonotic disease systems do not satisfy the listed prerequisites, but rather that the prerequisites lend themselves to the predictions of the dilution effect. That is, the decrease in the relative abundance of highly competent hosts is both necessary for the system to demonstrate a dilution effect while also being the predicted outcome of the hypothesis. For example, consider a vector‐borne disease system that satisfies all four prerequisites: (1) generalist vector, (2) horizontal pathogen transmission, (3) host competence variation, and (4) high competence hosts are also abundant in the community. Testing for a dilution effect in such a disease system would *always* result in evidence for a dilution effect, never evidence against. In these instances, we should be critical about how these hypotheses are being tested and if they continue to be useful in furthering our understanding of diversity‐disease relationships.

While conceptual frameworks and interaction webs are useful for generating hypotheses, observing a relationship between host diversity change and *overall* transmission does not lend support for any specific mechanistic pathways occurring. In other words, the dilution and amplification effects, when represented as *net* effects, cannot be tested without first testing their individual components. Others have implied this idea by advocating for a more mechanistic approach to diversity‐disease studies (Glidden et al. 2021; Halsey 2018; Johnson et al. 2015; Keesing and Ostfeld 2021; Kilpatrick, Salkeld, et al. 2017; Rohr et al. 2020; Stewart Merrill and Johnson 2020) and some have explicitly tested systems this way (Gandy et al. 2022; Kocher et al. 2023; Strauss et al. 2015). This issue of testing the global effect and backward inferring potential mechanisms that could be responsible for the observed pattern appears in several prominent diversity‐disease studies (Allan et al. 2003, 2009; Brownstein et al. 2005; Ezenwa et al. 2006; Ostfeld and Keesing 2000a) that have suggested support for dilution or amplification effects. These studies are important to help unveil patterns observed in natural systems, but results should not be extrapolated further if component effects have not been explicitly tested. Essentially, patterns are not unequivocal evidence of underlying processes and should not be interpreted as such (Cooper 1998). Caution toward this type of causal inference is not novel (Anderson 2008), and the same principle also applies in the opposite direction of interpretation, where evidence for one pathway does not necessarily represent the overall net pattern in the system when other pathways are not observed or accounted for.

Reviews of the dilution and amplification effect framework ultimately have come to contradictory conclusions regarding the strength (Civitello et al. 2015; Salkeld et al. 2013), directionality (Wood et al. 2017), and nature of the relationship (Halliday et al. 2020; Halliday and Rohr 2019; Johnson et al. 2015; Wood et al. 2017). Context dependencies exist to potentially dampen the observation of these effects, and, additionally, comparisons between studies (Rohr et al. 2020; Salkeld et al. 2013), which leads us to believe that the field should focus its attention on a priori approaches to these dependencies and their mechanisms instead of *ad hoc* justifications for contradictory evidence (Box 2). The latter may be doing more harm than good when dilution and amplification frameworks are continuously being modified to explain inconsistencies in the emerging data.

BOX 2 **Falsifiability**

A falsifiable hypothesis is a hypothesis that can be empirically tested and can obtain an observation that disproves the claim (Popper 2002). Factors that make a hypothesis non‐falsifiable can include issues with specificity (e.g., too vague such that specific predictions are not possible, undefined variables with no clear relationship between them), issues with testing the hypothesis itself (e.g., impossible/supernatural observations, undefined metrics), or issues with the reasoning behind the hypothesis (e.g., circular reasoning, unbounded scope).

**Ad Hoc Explanations**

An *ad hoc* explanation occurs when modifications are made to a theory that result in an evasion from falsification. These modifications may involve introducing *ad hoc* auxiliary hypotheses (i.e., assumptions or prerequisites to a theory), *ad hoc* changes to definitions, and *ad hoc* explanations for inconsistencies in new evidence that would prevent the original hypothesis from being falsified (Popper 2002). In this case, the hypothesis effectively becomes too flexible to be tested rigorously.

**Null Hypothesis**

A null hypothesis is the outcome that describes no effect or relationship between the variables being tested. A good null model considers the “default” system for the relationship being tested (i.e., null predictions should be informed by historical data to create a baseline rather than assuming no relationship exists in the first place). For example, Rohr et al. (2011) critique the null assumptions about host–parasite phenological mismatches in testing climate‐disease models, which assume perfect synchrony when historical evidence suggests otherwise. In disease ecology studies, specific hypotheses help identify what transmission pathway is being tested, and in turn, what the null model would look like when no such pathway is operating. Whereas the null model for the net effect of biodiversity on disease transmission, more broadly, is less clear due to the ambiguity of this hypothesis and the variations between the disease systems it could be applied to.

The aim of this paper is to evaluate the traditional net dilution and amplification effect framework through a critical lens. In particular, we ask (1) are these hypotheses falsifiable?; and if so, (2) how should we test them? We further demonstrate our claims using a case study in the Lyme disease (LD) system through a systematic review of the literature. To conclude, we discuss implications for future diversity‐disease research more broadly and in the context of applications in disease management.

## 3 Hypotheses Should Be Unconditionally Falsifiable

Controversy surrounding the dilution and amplification effects has historically focused on inconsistencies in how we define disease risk and measure transmission, leading to variable results and overall misconceptions about these hypotheses (Huang et al. 2016; Johnson et al. 2015; Keesing and Ostfeld 2021; Kilpatrick, Salkeld, et al. 2017; Randolph and Dobson 2012; Rohr et al. 2020). For instance, disease risk, typically measured as the abundance (i.e., density) or prevalence (i.e., proportion) of infected individuals, has been conflated with parasite diversity (i.e., richness) where the latter does not represent the pathogen transmission per se but rather pathogen establishment or colonization (Johnson et al. 2013, 2015). While parasite diversity is not entirely irrelevant to the diversity‐disease relationship, it is incorrect to suggest that evidence for host diversity correlating positively with parasite diversity (Dunn et al. 2010; Kamiya et al. 2014) is also evidence in support of the amplification effect. Similarly, if our abundance and prevalence metrics do not equally measure pathogen transmission, which has been suggested to be the case (Huang et al. 2016; Randolph and Dobson 2012), they are less comparable. These issues are evident and pose valid criticisms regarding the clarity of these hypotheses; however, we suggest that a philosophical problem also exists in its foundation: dilution and amplification *net* effects are not falsifiable hypotheses.

### 3.1 Non‐Falsifiable Hypotheses

At its core, a hypothesis is a scientific model that can offer support for a phenomenon if it can also be rejected through testing (Box 2). The net effect hypotheses proposed by the dilution and amplification effects cannot be falsifiable if an observed null outcome, implying no net relationship between host diversity and disease transmission, can also be explained by a canceling out of opposing component transmission mechanisms. In other words, the absence of a net effect is not necessarily explained by a true null. When this is the case, the hypothesis cannot be appropriately falsified with a null outcome. An example of counteracted effects can be observed in the LD system, where western fence lizards ( *Sceloporus occidentalis* ) are important blood meal hosts for tick vectors, while also being non‐competent hosts for the pathogen. As a result, these two opposing effects, vector amplification (i.e., increasing the abundance of tick vectors in the community) and pathogen dilution (i.e., decreasing the average host competence in the community), cancel out in experimental removals of *S. occidentalis* to produce a net null effect on vector infection prevalence (Swei et al. 2011). This lack of overall effect does not tell us that *S. occidentalis* removal (i.e., decreased host diversity) had no effect on LD risk, but rather that these component mechanisms can independently amplify and dilute transmission. Despite the fact that *S. occidentalis* does indeed dilute the prevalence of infection, this role can be overlooked when only the net effect is considered. Additionally, when disease risk is alternatively measured as the density of infected vectors, risk decreases with *S. occidentalis* removal, contrary to the assumptions of a dilution effect whereby a loss of non‐competent hosts increases disease risk (Swei et al. 2011). This contradiction may initially mischaracterize the underlying mechanisms in the system in the same way an observed null would. In this example, lizards play a unique role in the LD system such that testing and interpreting the net dilution and amplification effect hypotheses at a broad scale (i.e., biodiversity loss overall or disease risk overall) does not account for these species‐specific variations in influencing disease transmission.

While it is possible to test a net effect and definitively observe a null relationship through aggregating known component effects, this also requires testing individual mechanisms as separate hypotheses to be included in a multivariate causal model (e.g., structural equation modeling). That the same pattern, a null outcome, can be explained by multiple processes (either a lack of relationship between biodiversity and disease risk, i.e., a true null, or a canceling out of amplification and dilution effects when mechanisms interact) means that the pattern of a null outcome sheds no light on the underlying process. Consider a meta‐analysis conducted to test the net dilution and amplification effects by Salkeld et al. (2013). They conclude that across multiple disease systems, the diversity‐disease relationship is inconsistent, with weak effect sizes that cancel out. This null outcome suggests that the cumulative effect of biodiversity on disease risk is not generalizable across different disease systems, while also not separating out the process that generated the overall pattern. In other words, the different disease systems are too context‐dependent to be generalized by an observed pattern, and any generalization would be of a pattern, not a process that drives the diversity‐disease relationship. If the individual studies included in a meta‐analysis are not explicitly testing the same transmission mechanism driving a diversity‐disease relationship, meta‐analyses including these studies are not truly as informative as they have been interpreted. A different meta‐analysis concluded a strong net dilution effect across multiple disease systems but acknowledged the inability of their meta‐analysis to test these mechanisms (Civitello et al. 2015). In these meta‐analyses, the presence or absence of a net effect is testable in this way; however, its component mechanisms are not. This is also meant to highlight the difficulties associated with testing a net effect in a simplistic bivariate model. It lacks the explanatory power of multivariate models, and we suggest that if one is to be conducted, future meta‐analyses should focus their attention toward testing the pooled effect of specific mechanistic relationships (Heckley and Becker 2023; Murray et al. 2019; Vicente‐Santos et al. 2023; Young et al. 2013). For example, such a mechanism in the LD system could be the effect of the relative abundance of highly competent *Peromyscus* spp. (deer mice) hosts on nymphal infection prevalence with *B. burgdorferi* (Bouchard et al. 2013; LoGiudice et al. 2008; Mason et al. 2022; Werden et al. 2014). This example would provide information about the strength of the amplifying potential of *Peromyscus* spp. in the LD system across studies, and hence, a specific mechanistic relationship that can be evaluated.

Additionally, the broadness of these net effect hypotheses also makes them vulnerable to *ad hoc* explanations of context dependencies (Box 2) by using the present context as an alternative scenario rather than as evidence already present for falsification. An a priori hypothesis that lacks specificity essentially loses some of its confirmatory nature as a hypothesis, as it opens up opportunities for the conflation between hypothesis testing (i.e., does the evidence support my hypothesis?) and *ad hoc* explanations of unexpected results (i.e., how could the evidence support my hypothesis?; Gelman and Loken 2013). While this type of *post hoc* reasoning can be arguably justified since it is often still theory‐driven and not spurious, it is also important to note that it also provides explanations of inconsistencies in the data that would otherwise have been evidence to falsify the original hypothesis (Popper 2002). For example, Ezenwa et al. (2006) observed a negative correlation between non‐passerine avian diversity (low‐competence hosts) and incidence of West Nile virus (WNV) in humans. On the other hand, they observed no correlation between passerine diversity (high competence hosts) and WNV incidence or mosquito infection rates, which would have been consistent with the first observation. Nonetheless, support for a dilution effect was claimed, despite conflicting evidence, by reasoning that these high competence species were more common in all sampling sites. To be clear, *ad hoc* explanations are a systemic issue, and we intend to only highlight these subtle lines of reasoning using common examples so as not to overlook them as a whole. The concern is that these *ad hoc* explanations may also lend themselves to a posteriori hypotheses which, at best, do not hold strong causal weight (Anderson 2008). In another example, Clay et al. (2009) observed that rodent species diversity correlated negatively with Sin Nombre virus prevalence in deer mice, suggesting a dilution effect. However, they also note that this outcome could not have been predicted a priori because the opposite relationship could have been observed. The use of a posteriori reasoning should be recognized as exploratory hypothesis generation rather than confirmatory evidence for a phenomenon. While exploratory hypotheses are not inherently disadvantageous (Rubin and Donkin 2024), we are concerned that they are being misinterpreted as confirmatory tests or not being adequately falsified.

The growing list of prerequisites for these dilution and amplification effect frameworks may also unintentionally encourage researchers to look for additional “missing pieces” to unify previously contradicting results regarding the nature of the diversity‐disease relationship (Halliday et al. 2020; Wood and Lafferty 2013). The modification of these prerequisites (“auxiliary hypotheses” *sensu* Popper 2002) is not inherently problematic, as the hypothetico‐deductive model of the scientific method follows this process of evaluating tested hypotheses to then modify and generate new testable hypotheses. However, this process also distinguishes between the old and new hypotheses as separate testable hypotheses through this process of modification, which should translate into a revision of the whole system (i.e., ‘strong inference’ Platt 1964). The *ad hoc* modification of auxiliary hypotheses, and indirectly the original hypothesis being tested, should be criticized when it preserves the integrity of the original hypothesis and theoretical framework by changing the conditions of falsification (Popper 2002). In the case of the dilution and amplification effects, any inconsistencies in the data from these studies should question the support for these net effect hypotheses themselves, in addition to their auxiliaries, rather than only the latter.

It is tempting to provide *ad hoc* explanations, especially in diversity‐disease studies when there is a clear motivation to want to generalize the dilution or amplification effects to different disease systems (Glidden et al. 2021; Halliday and Rohr 2019; Keesing and Ostfeld 2021; Ostfeld and Keesing 2012; Rohr et al. 2020). In the case of the dilution effect specifically, there is a vested interest embedded in finding support for this hypothesis, considering how the “biodiversity‐buffers‐disease” paradigm (Randolph and Dobson 2012) aligns with the goals of conservation biology. This ‘win‐win’ scenario is certainly appealing, but caution should be applied such that this motivation does not lead to the adoption of theories for solely practical reasons. Popper (2002) refers to the idea of conventionalism to highlight the dangers of dogmatism in science, especially when theories remain unfalsifiable as a result of the more insidious practice of *ad hoc* reasoning as opposed to outright denial. To borrow from another philosopher of science, Kuhn (1962) notes that only when the old guard of ideas have been discarded can there be a scientific paradigm shift. In this instance, the traditional net effect approach to testing dilution and amplification effects should be re‐evaluated in favor of a mechanistic approach to investigating the diversity‐disease relationship.

### 3.2 Testing Mechanistic Pathways

Rather than observing an overall pattern and potentially overlooking underlying interactions, identifying evidence in support of specific disease transmission pathways (i.e., ‘component effects’) is crucial for piecing together the bigger picture in the diversity‐disease relationship. On the other hand, it is equally important to falsify our initial hypotheses when observing a null relationship instead of generating *ad hoc* explanations as to why amplification or dilution might still be acting. Clarity and precision in hypothesis generation will allow us to test specific hypotheses in a system, acting as a shield against *ad hoc* explanations. By developing these hypotheses a priori (confirmatory) as opposed to continuing to test the whole system and generating a posteriori hypotheses (exploratory), we will streamline the progress in disease ecology research and theory development (Anderson 2008; Grace 2006). This is especially important to parameterize predictive and causal models, which is another goal in disease ecology (Johnson et al. 2015; Rohr et al. 2020), by supplying data about the component effects in the system. As mentioned previously, net effects are testable in this sense if component mechanisms are clearly defined with measurable effect sizes to reflect the proposed transmission drivers in a system. Methods such as structural equation modeling (SEM) to test a priori hypotheses in a theoretical framework would be particularly useful in this case, where we are essentially examining how a network of direct and indirect transmission drivers influences the overall diversity‐disease relationship (Clark et al. 2019; Fearon et al. 2023; Figueroa et al. 2020; Grace 2006; Grace et al. 2014; Laughlin and Grace 2019; Shipley 2000). In this way, we can definitively test and come to conclusions about the existence of any suppressed (i.e., canceled out) or offsetting (i.e., mediated) effects that are overlooked in net effect hypotheses. See Laughlin and Grace (2019) for a more detailed discussion on these effects in the context of SEMs.

**FIGURE 2:**
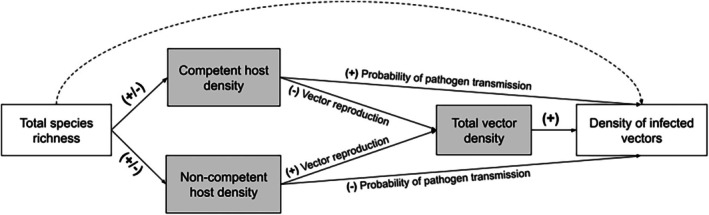
Path diagram depicting a hypothetical vector‐borne disease system where the effect of biodiversity (total species richness) on disease risk (density of infected vectors) is mediated by host competence (competent or non‐competent) and total vector density (gray boxes). Total vector density is also influenced by the competence of the host, such that competent hosts are also poor reproductive hosts for the vectors and vice versa. Each path represents a proposed causal path between variables with the relationship *β*. Signs on the direct paths (solid arrows) between variables show the directionality of the relationship between them (positive, negative, or either). The net indirect effect between total species richness and density of infected vectors (dashed arrow) is the product of the coefficients along all component paths (Wood and Lafferty 2013).

For example, consider a simple two‐host vector‐borne disease system where biodiversity is measured as total species richness, and disease risk is measured as the density of infected vectors (Figure 2). The effect of increasing species richness is mediated by the relative abundances of competent and non‐competent hosts (+/− probability of pathogen transmission), in addition to vector density, which is also influenced by host competence in this system (+/− vector reproduction). Similar to the example with western fence lizards (Swei et al. 2011), the relationship between a host's competence and its ability to serve as a reproductive host for the vector is inversely related in this particular system. The diluting effect of decreased pathogen transmission with an increase in non‐competent hosts is canceled out by the amplifying effect of increased host‐vector contact overall, as a result of increased total vector density. The net outcome would see a null or much weaker relationship between total species richness and density of infected vectors (dashed arrow). Through this SEM path diagram, we are able to observe these mediators canceling out the diluting and amplifying effects of different hosts in the system.

Partitioning our net effect hypotheses into smaller mechanistic hypotheses also addresses practical issues with defining and measuring disease risk. While the hypotheses proposed by Ostfeld and Keesing (2000b) explicitly reference the use of disease prevalence metrics (e.g., proportion of infected vectors or hosts), the use of disease abundance metrics (e.g., density of vectors or hosts) has been a common representation of disease risk (Huang et al. 2016; Wood and Lafferty 2013). This problem not only creates difficulties when comparing results across studies, but also affects which net effect hypothesis, dilution, or amplification is supported, depending on the reference measurement (Gandy et al. 2021; Huang et al. 2016). These contradictory net effect results leave more questions unanswered regarding the underlying interactions between transmission mechanisms and fail to capture the complexity associated with measuring abstract variables with multiple proxy metrics.

We propose that biodiversity and disease risk should be treated as unobserved latent variables rather than directly measured variables (Box 3). In this way, we can visualize the different ways that biodiversity and disease risk can be indirectly measured through proxy metrics in a latent variable model.

**FIGURE 3:**
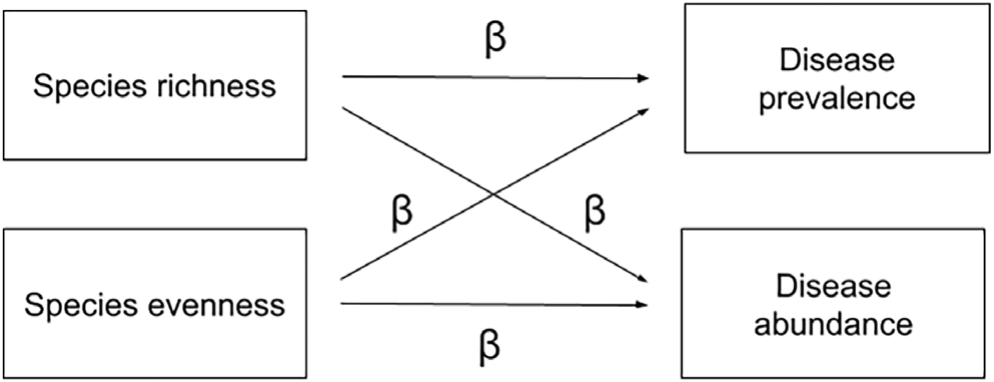
Observed variable model of a simplified diversity‐disease relationship that includes only measured variables. Observed variables (species richness, species evenness, disease prevalence, and disease abundance) are represented as rectangles. Single‐headed arrows represent proposed causal paths between variables, with *β* representing the standardized or unstandardized bivariate regression coefficients.

BOX 3 Structural equation modeling (SEM) is a useful statistical technique to model casual relationships between intercorrelated variables (Grace 2006; Laughlin and Grace 2019; Shipley 2000). There are two models within the SEM framework that can be applied in this context to conceptualize these relationships: (1) an observed variable model and (2) a latent variable model. We can model component mechanistic relationships as paths between observed variables (i.e., directly measured variables) in an observed variable model (Figure 3).

**FIGURE 4:**
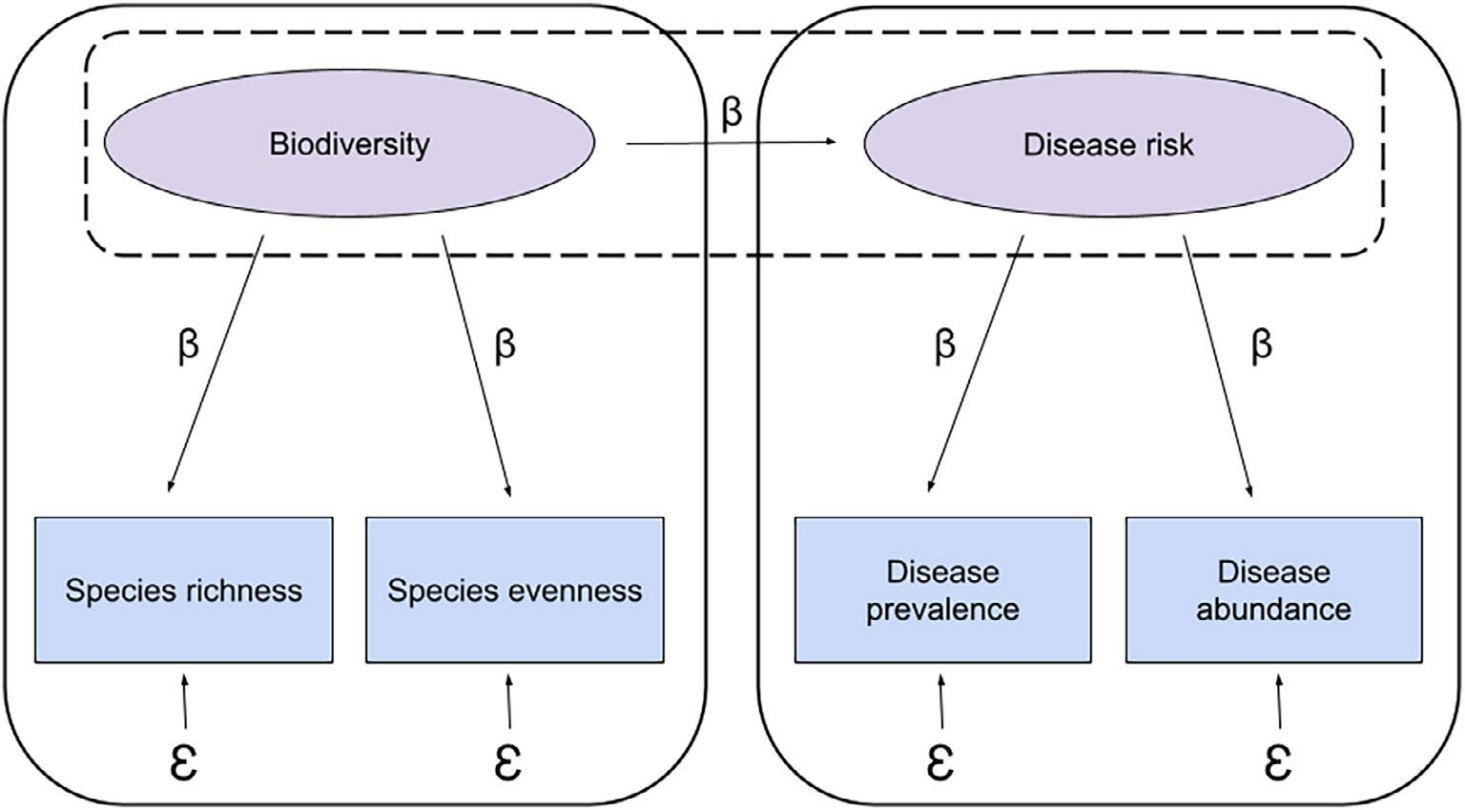
Latent variable model of the diversity‐disease relationship with two components: (1) a structural model describing the relationship between latent variables (dashed black outline) and (2) a measurement model for individual latent variables (solid black outline). Unobserved latent variables (biodiversity and disease risk) are represented as ovals and observed measured variables (species richness, species evenness, disease prevalence, and disease abundance) are represented as rectangles. Single‐headed arrows represent proposed causal paths between variables, with *β* representing the standardized or unstandardized bivariate regression coefficients. *Ɛ* represents error, including measurement error and error that is introduced via stochasticity in the system (i.e., irreducible error).

We can further expand on this observed variable model by representing correlated variables as proxies represented by a shared unobserved latent variable (i.e., indirectly measured construct) in a latent variable model (Figure 4).

**FIGURE 5:**
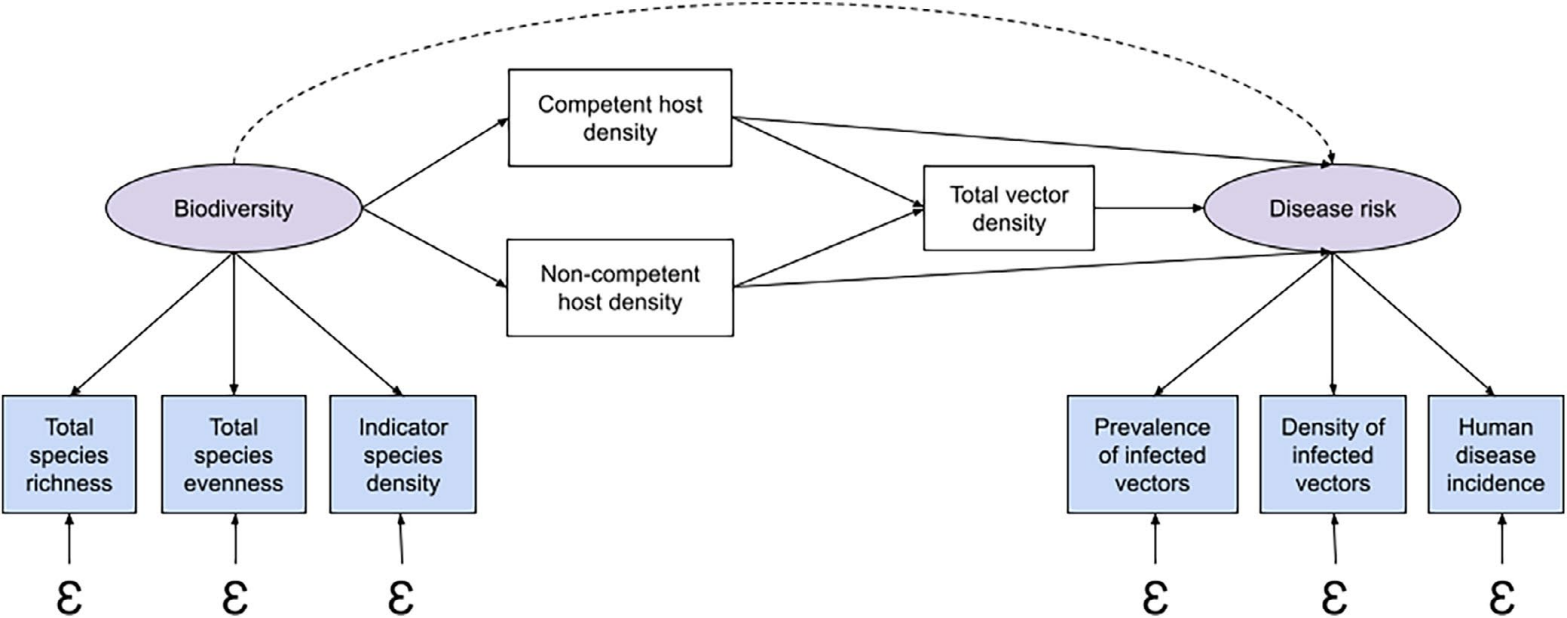
Generalized latent variable model of the proposed path diagram in Figure 2 (relationships not specified). Unobserved latent variables are represented as ovals and observed measured variables are represented as rectangles. Mediator variables in the relationship between biodiversity and disease risk are represented as white rectangles. Proxy metrics representing the latent constructs are represented as blue rectangles. Direct paths are represented by solid arrows and the net indirect effect is represented by a dashed arrow. Each path represents a proposed causal path between variables with the relationship *β*. The net indirect effect (dashed arrow) is the product of the coefficients along all component paths. A positive *β* on the net indirect effect would indicate a net amplification effect, whereas a negative *β* would indicate a net dilution effect. A zero *β* would indicate a net null effect of biodiversity on disease risk. *Ɛ* represents error, including measurement error and error that is introduced via stochasticity in the system (i.e., irreducible error).

Latent variable models incorporate a structural model that consists of paths between latent variables. In this case, the path between biodiversity and disease risk represents the hypothesized causal relationship between these two latent variables, which are indirectly measured. Mechanistic relationships are also represented by these paths, as was the case in the observed variable model, but the latent variable model makes a distinction between theoretical concepts and proxy metrics being used (Grace 2006; Shipley 2000). Additionally, the path between the latent variables can include mediator variables in the same way a path analysis would, providing a more complete picture of the diversity‐disease relationship as one that is indirect and conceptual. For example, we propose the path diagram in Figure 2 can be modified to incorporate a measurement model into the existing structural model (Figure 5).

Our simplified SEM can be generalized to many zoonotic disease systems where the diversity‐disease relationship is being tested. While our model is a generalized vector‐borne system, Figure 5 can be modified to include mediator variables (white rectangles), proxy metrics (blue rectangles), and bivariate relationships (solid arrows) that are specific to any zoonotic disease system where biodiversity is proposed to influence disease transmission. Evidence for a bivariate relationship (i.e., a significant non‐zero *β* coefficient along a solid arrow path) would provide evidence for that component mechanism. For example, a positive *β* between non‐competent host density and total vector density would indicate that non‐competent hosts exhibit an amplifying effect on disease risk through the increase in vector density (assuming total vector density is also positively linked to disease risk). Should the product of all coefficients along the component paths between biodiversity and disease risk (i.e., the net indirect path) also be non‐zero and significant, evidence for a net relationship between biodiversity and disease risk would be present. While our SEM only models biotic interactions, abiotic variables, such as habitat, can also mediate diversity‐disease relationships and are worthy of consideration and can be incorporated the same way (Fearon et al. 2023; Figueroa et al. 2020).

When component mechanisms are tested in this way, multiple metrics for biodiversity and disease risk can also be tested and assessed for validity in the measurement model. In other words, we can assess how representative our proxy metrics are of the intended latent construct. Additionally, these multi‐indicator models lead to more accurate estimates of the latent construct and greater generalizability of our causal model (Grace 2006; Shipley 2000). The advantage of latent variable models is that they consider the reliability of these metrics to estimate measurement error, which is inherent in proxy measures. Large amounts of measurement error, indicated by low reliability estimates, can also suggest changes to measurement protocols that may be warranted for future studies (Lamb et al. 2011). As there is still discussion about the best metric to represent biodiversity and disease risk (Huang et al. 2016; Randolph and Dobson 2012), especially from a practical conservation and human health perspective (Hopkins et al. 2022; Kilpatrick, Salkeld, et al. 2017; Wood et al. 2017), modeling these relationships in a latent variable model can be useful for researchers to synthesize multiple metrics together and gain clearer insight into the underlying relationships between measured and latent variables within the broader diversity‐disease picture.

However, limitations to this approach include model misspecification. For example, if there is a theoretical reason to believe that the proxy metrics (prevalence of infected vectors, density of infected vectors, and human disease incidence) are not all represented by the latent construct denoted as disease risk (Figure 5), they should not all be included in the model. Similarly, empirical considerations include known measurement error and reliability estimates of the proxy metrics that may suggest the metrics are not suitable estimates for the model (Fan et al. 2016; Grace et al. 2010). In these instances, a smaller subset of the proxy metrics that represent key facets of the latent construct (i.e., not highly interchangeable) may be preferred to generate a more parsimonious model. Other preliminary methods, such as confirmatory factor analysis, may be utilized to first test the relationship between latent variables and their measured proxies (Fan et al. 2016; Shipley 2000).

## 4 A Case Study in Lyme Disease

We present a case study using the vector‐borne LD system to demonstrate that the amplification and dilution effect hypotheses are not falsifiable and require re‐evaluation. LD is a common and widespread tick‐borne disease in temperate regions of North America, Europe, and Asia, with a well‐understood zoonotic cycle and ecology (Kurtenbach et al. 2006). The *Ixodes* tick life cycle is characterized by four developmental stages: egg, larva, nymph, and adult that involve three blood meals (larval, nymphal, and adult stages) where the causative bacterial agent, *Borrelia burgdorferi* , can be either transmitted from host to tick (all post‐egg stages) or tick to host (nymphal and adult stages only). Generalist *Ixodes* tick vectors feed on hosts that vary in *B. burgdorferi* competence, where smaller mammals tend to be the most competent and abundant hosts in the community (Barbour et al. 2015).

The frequency‐dependent nature of *B. burgdorferi* transmission, reliant on the proportion of competent hosts available to infect *Ixodes* tick vectors, would suggest a relationship between host diversity and disease risk, prompting studies investigating the amplification and dilution hypotheses in this system. Historically, the dilution effect has been the accepted hypothesis in the LD system when studied in endemic regions of the Northeastern United States (Allan et al. 2003; LoGiudice et al. 2003; Ostfeld and Keesing 2000a). However, there has been evidence to suggest that the observed dilution effect exists only under specific circumstances of community composition and landscape scale, garnering criticisms about its robustness and generalizability across other regional LD and zoonotic disease contexts (Kilpatrick, Dobson, et al. 2017; MacDonald et al. 2022; Randolph and Dobson 2012; Salkeld et al. 2013; Salkeld and Lane 2010; Wood and Lafferty 2013). In particular, *B. burgdorferi* sensu lato is vectored by different *Ixodes* spp. in its global distribution, with a large repertoire of hosts that vary regionally. As a result, the ecological context between regions varies in their species compositions and temporal transmission dynamics (Kurtenbach et al. 2006).

There has been a trend of increasingly mixed support for the dilution effect within the LD literature that continues to be largely unresolved, which may indicate that the traditional net effect framework is no longer useful in determining the role of biodiversity in influencing *B. burgdorferi* transmission. The framework permits too much flexibility in our interpretation of results, while not actively testing for nuance in the system. We conducted a systematic review of the LD literature to evaluate how these dilution and amplification effects are being tested and the general consensus on the directionality of the diversity‐disease relationship. We suggest that the past approach to investigating these effects be reconsidered, given the current disconnect in the literature as a result of the context dependencies that exist.

## 5 Methods

### 5.1 Database Search

**FIGURE 6:**
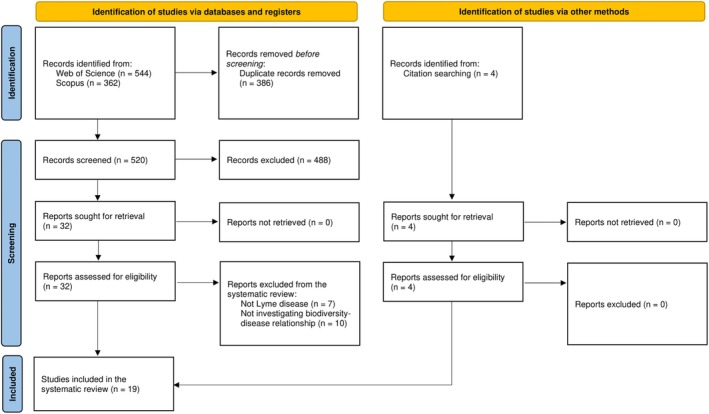
A PRISMA flow chart outlining the inclusion process for the systematic review (Page et al. 2021). Reports assessed for eligibility (*n* = 32) from the database searches include those that underwent abstract and full‐text screening due to a large number of reports being excluded (*n* = 488) during title screening. From the reports assessed for eligibility from the database searches (*n* = 32), 15 were included. Total studies included in the systematic review (*n* = 19) include the addition of eligible reports from citation searching (*n* = 4).

We conducted a systematic search following the Preferred Reporting Items for Systematic Reviews and Meta‐Analyses (PRISMA) guidelines (Moher et al. 2009). Using the Web of Science and SCOPUS databases (1900–Present), we conducted the original search string: “mammal* AND tick* AND disease* AND (dilut* OR amplif* OR biodiversity OR diversity OR richness OR evenness) NOT (livestock OR domestic*)” (November 2023). We obtained a total of 447 papers after removing duplicates. We conducted another search due to a low output of final papers in the original search string with an updated search string: “mammal* AND lyme AND (dilut* OR amplif* OR biodiversity OR diversity OR richness OR evenness) NOT (livestock OR domestic*)” (December 2023) to obtain an additional 73 papers after removing duplicates. We conducted additional citation searches to further obtain 6 papers for a total of 526 unique papers overall. A total of 38 papers were eligible for assessment (out of 526) after conducting a systematic search of the literature, with 19 included in the systematic review (Figure 6).

### 5.2 Selection Criteria

For the systematic review, we included papers if they tested the diversity‐disease relationship in LD explicitly, with respect to *Ixodes* tick hosts of *B. burgdorferi* (*n* = 19). We included both empirical and simulation studies with no restriction on which biodiversity or LD risk metric was used. Some papers that we included in the systematic review included multiple separate analyses of the diversity‐disease relationship using different parameters. As a result, we categorized each analysis (*n* = 30) within each paper (*n* = 19) by the study type (empirical or simulation), the biodiversity metric used, the LD risk metric used, and the outcome (dilution, amplification, or none). Studies that measured biodiversity using site features such as site size or habitat type (interior vs. edge) were categorized as “site proxy” under their biodiversity metric.

Some analyses were then grouped into broader host biodiversity and LD risk metric categories. Primarily, analyses that measured host biodiversity using any combination of species richness and evenness (e.g., Shannon Diversity index) were categorized as “combined indices”. Analyses that measured LD risk using any type of prevalence metric (e.g., nymphal infection prevalence (NIP), adult tick infection prevalence, or host infection prevalence) were categorized as “LD prevalence”. Analyses that measured LD risk using density of infected nymphs (DIN) were categorized as “LD abundance” for consistency and to highlight the categorization of ‘abundance’ and ‘prevalence’ metrics in the literature. Analyses that measured LD risk using density or total number of feeding or questing ticks were categorized as “tick burden”. For each outcome category (dilution, amplification, or none), we calculated the proportions for each biodiversity and LD risk metric used (i.e., proportion of analyses that used that metric) to show any trends within each outcome.

## 6 Results

**FIGURE 7:**
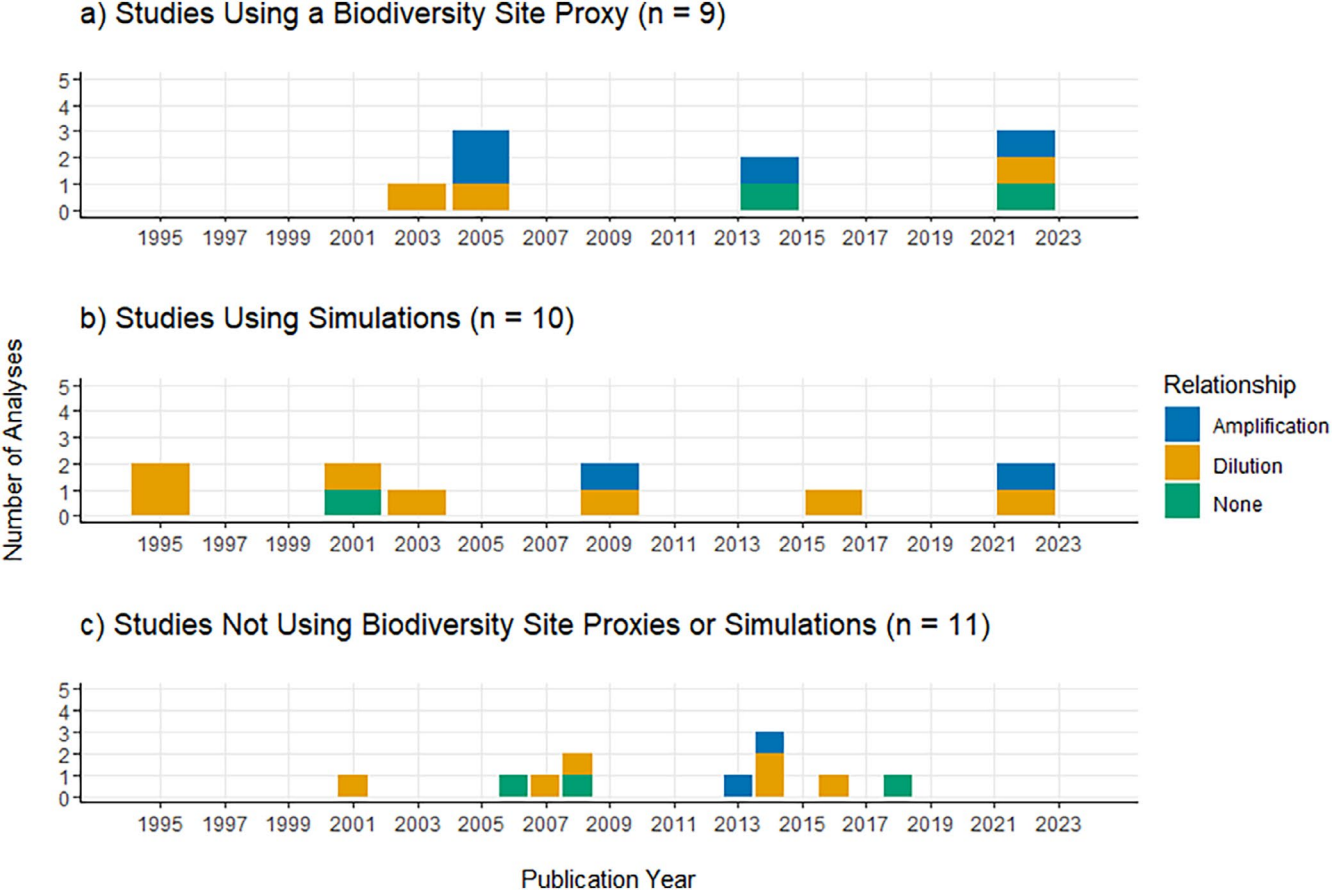
Proportional bar plots showing studies that use (a) site proxies (site size or habitat type), (b) simulations in analyses of the diversity‐disease relationship for LD over time. (c) represents studies that did not use site proxies or simulations (i.e., were otherwise empirical studies that used direct biodiversity metrics). The color‐coded proportions represent the outcomes (amplification [blue], dilution [orange], none [green]) for each analysis.

Most analyses overall use host richness as their biodiversity metric (*n* = 16) and LD prevalence (specifically NIP) as the LD risk metric (*n* = 9). Within analyses that found an amplification effect (*n* = 8), richness and site proxies were used equally, and LD abundance (i.e., DIN) was mostly measured as the LD risk metric. Within analyses that found a dilution effect (*n* = 16), richness was used the most, and LD prevalence was mostly measured. Within analyses that found no effect (*n* = 6), combined indices and site proxies were used equally, and LD prevalence was the typical measurement (Table 1). Overall, a little under a third of all analyses use site proxies as their biodiversity metric (*n* = 9) and found mixed evidence for the diversity–disease relationship. Another remaining third of studies were simulations that did not use a site proxy (*n* = 10) and mainly found evidence for a dilution effect. Among the studies that did not use a site proxy and were not simulations (*n* = 11), evidence was again mixed (Figure 7).

**TABLE 1:** Proportion of individual analyses (*n* = 30) from studies (*n* = 19) in the literature suggesting an amplification, dilution, or no effect of biodiversity on LD risk.

Metric	Amplification	Dilution	None
Host biodiversity			
Species richness	50.0%	68.8%	16.7%
Species evenness	0%	0%	16.7%
Combined indices	0%	12.5%	33.3%
Site proxy	50.0%	18.8%	33.3%
LD risk			
LD abundance	37.5%	18.8%	33.3%
LD prevalence	25.0%	50%	66.6%
Tick burden	25.0%	18.8%	0%
Human incidence	12.5%	12.5%	12.5%

*Note:* Within each outcome, the proportion of different biodiversity metrics and LD risk metrics measured is shown. Within host biodiversity metrics, “combined indices” refers to metrics combining species richness and evenness (e.g., Shannon Diversity index), and “site proxy” refers to site feature metrics (e.g., site size, habitat type). Within LD risk metrics, “LD abundance” refers to DIN (density of infected nymphs) and “LD prevalence” refers to NIP (nymphal infection prevalence), adult tick infection prevalence, or host infection prevalence. “Tick burden” refers to the density or total number of feeding or questing ticks. “Human incidence” refers to the incidence of LD in human populations.

## 7 Discussion

### 7.1 Latent Variable Problem

As discussed previously, differential conclusions regarding the diversity‐disease relationship have been due in part to the usage of different reference metrics. Huang et al. (2016) highlight this problem in studies on vector‐borne disease systems more broadly to criticize the ambiguity of the dilution effect. In this case, the driving mechanism for the dilution effect proposed by Ostfeld and Keesing (2000b) (i.e., increasing host diversity) does not account for an increase in total vector abundance in these systems, due to an overall increase in feeding opportunities that occurs concurrently with a decrease in infection prevalence with feeding diversion. These two ‘abundance’ and ‘prevalence’ metrics to measure disease risk are both used commonly in LD research (Wood and Lafferty 2013). In our review of the literature, most analyses that observed an amplification effect measured disease risk as LD abundance (DIN), whereas most analyses that observed a dilution effect used LD prevalence (NIP, adult tick infection prevalence, host infection prevalence; Table 1). This pattern suggests that in the LD system, ‘abundance’ and ‘prevalence’ metrics may not represent the same transmission pathway and should not be interpreted or used interchangeably. In particular, ‘abundance’ metrics, representing the proportion of infection, better represent the effect that increasing host diversity has on the likelihood of an *Ixodes* tick vector parasitizing a competent host and thus infecting it with *B. burgdorferi* . However, this metric is independent of the overall ‘abundance’ (i.e., density) of *Ixodes* vectors in the system, which can be influenced by other mechanisms in the system that are not accounted for by ‘prevalence’ metrics. If the goal of the traditional dilution and amplification effect framework is to test the net effect, these considerations should be made. Otherwise, a net effect approach is not appropriate when different metrics are being used across studies. In other words, inconsistent metric usage makes it difficult to dissect out any true underlying diversity‐disease relationship from the noise created as a result of differences in measuring the same construct.

While we cannot know if single metrics being reported are due to preferential reporting, studies that evaluate and report multiple metrics paint a clearer picture of the overall relationship and can help visualize instances of multiple pathways concurrently increasing and decreasing disease risk (Gandy et al. 2021, 2022; Kocher et al. 2023). Insight into how these component effects may cancel out allows us to also make more informed interpretations and concrete evidence for falsification regarding a null or weak effect in these multivariate analyses. Should it be the case where we standardize our metrics, evaluating the relationship will become more clear. However, as discussed previously, there is disagreement on the most representative metric (Hopkins et al. 2022; Huang et al. 2016; Kilpatrick, Salkeld, et al. 2017; Randolph and Dobson 2012; Wood et al. 2017). In essence, there is a problem with poor or incomplete representation of the latent variables, biodiversity, and disease risk, in diversity‐disease studies. As mentioned previously, these constructs should be treated as conceptual and not directly measurable, which is consistent with how these variables are already being measured through proxies. When biodiversity and disease risk are only being partially represented (i.e., through single metrics), this obscures our ability to make comparisons across studies that come to contradictory conclusions because we are restricted to making comparisons across the same metric.

### 7.2 Context Dependencies

One major criticism regarding the generalization of the dilution effect in LD focuses on the assumptions made about the system that would suggest the effect to be present. In a way, the dilution effect hypothesis uses overall host diversity as a proxy for the proportion of highly competent hosts when assuming a negative correlation (Ostfeld and Keesing 2000b). For example, *Peromyscus leucopus* (white‐footed) mice are commonly regarded as the primary reservoir host of *B. burgdorferi* in Eastern North America due to their high competence, abundance, and conspicuousness. In depauperate communities, *P. leucopus* is expected to persist, which contributes to their amplification potential as hosts in low species diversity settings (Ostfeld and Keesing 2000a). However, inconspicuous and scarcely studied reservoir host populations of *Blarina brevicauda* (Northern short‐tailed) and *Sorex cinereus* (masked) shrews have been identified as being equally, if not more, important predictors of LD risk (Brisson et al. 2008). This claim threatens one of the existing assumptions of the dilution effect hypothesis, that the most competent host should be the most abundant in depauperate communities (Ostfeld and Keesing 2000b), and introduces sampling biases when testing the framework. Similarly, the use of site proxies assumes that with increasing site size and at habitat edges, host diversity increases (Allan et al. 2003; Brownstein et al. 2005). These assumptions do not always hold true, as the lack of a relationship has been observed (Linske et al. 2018; Mason et al. 2022), which means the true relationship between host diversity and LD risk could differ from what has been reported in the literature when using site proxy measures. As a result of a significant proportion of studies using site proxies when measuring biodiversity (Figure 7), we advise caution in interpreting the results of these studies, especially when it is tempting to attribute the effects of site features (e.g., site size, habitat type) to the effects of host diversity on disease transmission. To echo Salkeld et al. (2013), this would result in an ecological fallacy—when data at a larger scale is used to infer features of its components. Furthermore, another central assumption that *I. scapularis* is equally likely to feed on any host it comes into contact with (i.e., a true generalist) is not necessarily the case (Ginsberg et al. 2021; Goethert and Telford 2022), which threatens the assumption about the vector's generalized host feeding patterns (Ostfeld and Keesing 2000b). For instance, Ginsberg et al. (2021) observed selective host choice in *I. scapularis* to favor low‐competence lizard hosts in the Southeastern United States compared to the Northeastern United States, where high‐competence mammalian hosts were preferred despite similar species compositions. The authors note that the mechanism behind this observed nonrandom host selection, whether it is driven by tick behavior or ecological factors that vary across latitude, is not known. However, what is clear is that the relationship between host diversity and *B. burgdorferi* prevalence is not solely dependent on the available proportion of competent hosts.

These are all examples of when assumptions function as auxiliary hypotheses to the dilution effect framework in the LD system, such that evidence that falsifies these auxiliaries should also weaken support for the primary framework. However, when interpreting unexpected results while testing the dilution or amplification effects, caution should be exercised so as to not make *ad hoc* suggestions that question the validity of auxiliary hypotheses as a substitute for falsifying the framework. For example, Werden et al. (2014) observed that the assumption that highly competent mice would dominate depauperate communities did not hold up in all of their sampled sites. However, they suggested that this represents the context‐dependent nature of the biodiversity–disease relationship. In this way, one of the auxiliaries can be falsified, but not the dilution effect itself. When we observe a null outcome of a net effect, it may not be clear which hypothesis lacks support in these frameworks that include multiple auxiliaries. As a result, it is increasingly important for auxiliaries to be tested independently and rigorously before including them in these dilution and amplification frameworks to allow for accurate interpretations of observations and falsification of these frameworks directly.

Additionally, a significant proportion of the diversity‐disease LD literature is reliant on simulation studies that are modeled off these assumptions about the system (Figure 7). Two of such assumptions necessary to produce a dilution effect in these models are (1) the decrease in the proportion of highly competent hosts when host diversity increases, and (2) an increase in tick feedings on low‐competence hosts due to increased contact rates (Ogden and Tsao 2009). Both assumptions have been falsified with empirical evidence (Ginsberg et al. 2021; Goethert and Telford 2022; Linske et al. 2018; Mason et al. 2022), which reduces the validity of these simulations in generalizing the dilution effect across all LD systems. If the dilution effect only holds in extremely specific circumstances, then it should not be considered a foregone conclusion that it will be observed. A null outcome must be considered in empirical studies. Simulations can be used to demonstrate the possibility of processes underlying patterns in empirical systems, but they cannot be used to justify the possible existence of patterns that have not been documented. Less specific models with relaxed assumptions produce both dilution and amplification effects, which further demonstrates the inconclusiveness of testing for net effects in this system under the current framework (Occhibove et al. 2022; Ogden and Tsao 2009).

### 7.3 A Proposed Multivariate Model for Lyme Disease

**FIGURE 8:**
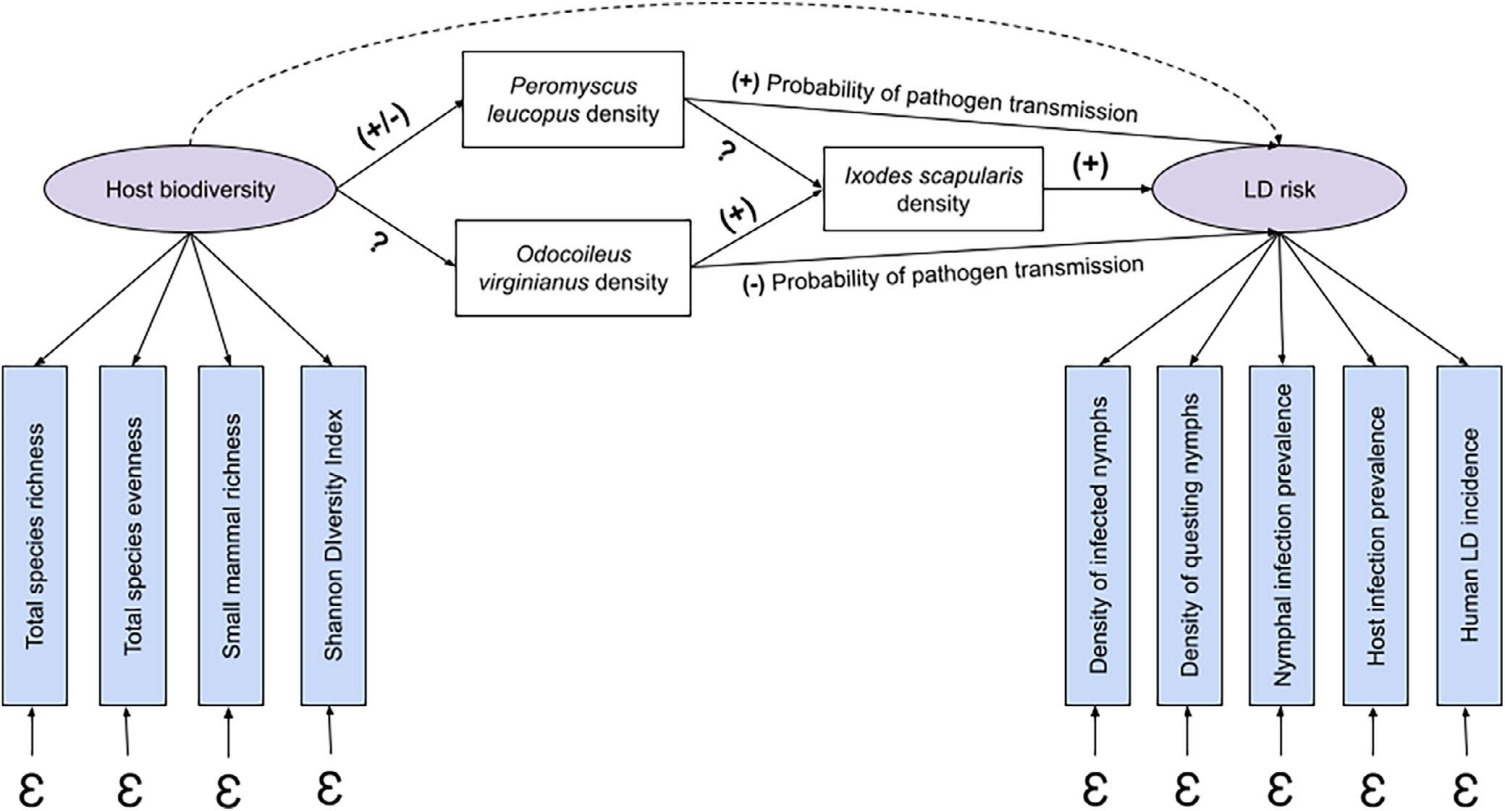
Latent variable model considering *Peromyscus leucopus* (white‐footed mice), *Odocoileus virginianus* (white‐tailed deer) density, and *Ixodes scapularis* (black‐legged tick) density as mediators in the relationship between host biodiversity and Lyme disease (LD) risk. Unobserved latent variables are represented as ovals and observed measured variables are represented as rectangles. Mediator variables in the relationship between biodiversity and disease risk are represented as white rectangles. Proxy metrics representing the latent constructs are represented as blue rectangles. Direct paths are represented by solid arrows and the net indirect effect is represented by a dashed arrow. Each path represents a proposed causal path between variables with the relationship *β*. A positive *β* on the net indirect effect (dashed arrow) would indicate a net amplification effect, whereas a negative *β* would indicate a net dilution effect. A zero *β* would indicate a net null effect of biodiversity on disease risk. Signs on the direct paths between variables show the directionality of the relationship between them (positive, either, or unknown) as evidenced in the literature (Elias et al. 2020; Kilpatrick et al. 2014; Linske et al. 2018; LoGiudice et al. 2003, 2008; Mason et al. 2022; Pepin et al. 2012; Werden et al. 2014). *Ɛ* represents error, including measurement error and error that is introduced via stochasticity in the system (i.e., irreducible error).

We suggest that SEM models can be used to test the diversity‐disease relationship in the LD system in a way that considers its latent variables and context dependencies. In doing this, we apply our proposed model in Figure 5 in the context of LD and include the host biodiversity and LD risk metrics summarized in Table 1, with the exception of site proxies because they are not causally predicted by host diversity (Figure 8).

We included two well‐studied hosts of *I. scapularis* in the Northeastern United States LD system, highly competent white‐footed mice ( *P. leucopus* ) and low‐competence white‐tailed deer ( *Odocoileus virginianus* ), to represent amplifying (+ probability of pathogen transmission) and diluting (− probability of pathogen transmission) pathways, respectively (LoGiudice et al. 2003; Figure 8). Additionally, we included *I. scapularis* density as a mediator, which positively predicts LD risk (Pepin et al. 2012) and is positively predicted by *O. virginianus* (Elias et al. 2020; Kilpatrick et al. 2014). The relationship between biodiversity and *P. leucopus* density is unclear, with studies seeing negative and positive associations (Linske et al. 2018; LoGiudice et al. 2008; Mason et al. 2022; Werden et al. 2014). The pathways between *P. leucopus* density and *I. scapularis* have not been identified in the literature, as well as the link between biodiversity and *O. virginianus* density, which presents an opportunity for future research to fill this gap. Additional gaps regarding our mechanistic understanding of how biodiversity can indirectly influence *B. burgdorferi* transmission can be worked into this model to build on what is already known from previous studies.

While our SEM includes only a few key mediators that influence the LD diversity‐disease relationship, we believe that it provides a starting framework for future LD studies that intend to synthesize together mechanistic pathways. It should be noted that our SEM broadly focuses on the biotic interactions characterizing the ecological context in the Northeastern United States LD system. The tick vectors for *B. burgdorferi* and host repertoire in the Western United States LD system differed significantly, with *Ixodes pacificus* (western black‐legged tick) and *Ixodes neotomae* maintaining transmission by feeding on highly competent dusky‐footed woodrats ( *Neotoma fuscipes* ). Where *P. leucopus* are common and highly competent in the Northeastern LD system, *Peromyscus* spp., namely *Peromyscus maniculatus* (eastern deer mouse), that are common in the Western LD system, are low‐competence hosts (Brown and Lane 1992; Swei et al. 2011). However, these differences can be more readily accounted for in SEM by modeling these context dependencies directly, whereas the traditional net effect approach would fall short of testing the diversity‐disease relationship in the Western LD system due to a violation of one of the prerequisites.

While our proposed SEM includes most of the host biodiversity and LD risk metrics from our systematic review, summarized in Table 1, one limitation is that not all of these metrics may be appropriate in the final model. As discussed previously during our introduction of SEM, theoretical and empirical considerations must be made during the model‐building process to avoid misspecification (Fan et al. 2016; Grace et al. 2010). Additionally, we can test the causal relationship between latent variables and their observed variables (i.e., proxy metrics) through methods such as confirmatory factor analysis to determine the validity of proxies being used in the current literature (Shipley 2000). Should we have the empirical data necessary to build this model, these proxy metrics can be properly evaluated to determine their contribution to the model. See Grace et al. (2010) for a more detailed discussion on these considerations when building SEMs in ecological contexts.

## 8 How Can Future Studies Improve Research on the Biodiversity–Disease Relationship?

A mechanistic focus on the diversity‐disease relationship entails a bottom‐up approach to investigating component mechanisms individually, as part of a larger network. Working to develop and test specific, a priori hypotheses that describe a clearly defined mechanism linking biodiversity to zoonotic disease transmission allows us to evaluate the effect of individual pathways and their interactions with other pathways. Additionally, it allows us to more readily accept that not all proposed mechanisms will be statistically or causally related to disease risk, which confers an advantage over testing the traditional net effect framework. We encourage future studies to test these mechanisms experimentally to demonstrate causality and mount stronger evidence to support diversity‐disease patterns occurring in nature (Venesky et al. 2014).

One argument for the practical usage of net effects is that they provide information about the cumulative increase or decrease of disease risk in a community, and as a result, we can gauge the net impact of biodiversity conservation interventions (Kilpatrick, Salkeld, et al. 2017). However, these net effects being a cumulation of multiple different pathways means that any given intervention can have potential trade‐offs. When these context dependencies exist in a system, there can be positive and negative effects on disease risk that occur simultaneously as a result of a change in biodiversity. Increasingly targeted strategies can minimize off‐target effects; however, these require robust evidence at the pathway level rather than that of a net outcome. This evidence gap in our mechanistic understanding and the inconsistency of these relationships under different circumstances hinders our ability to translate diversity‐disease research into practice when the risks outweigh the potential benefits (Hopkins et al. 2022). Our approach encourages the synthesis of multiple mechanisms in multivariate models to consider bivariate relationships in the context of their interaction with others. In this way, we can test for the unique contribution of biodiversity when other confounds are controlled for (Laughlin and Grace 2019). It is important not to conflate the role of decreasing biodiversity with other anthropogenic influences, such as land‐use change, which affect zoonotic disease transmission (White and Razgour 2020), even independently of biodiversity change (Wood et al. 2017). Furthermore, in proposing biodiversity and disease risk as latent variables, we can be more precise with how we represent these constructs to avoid blanket conclusions informing disease management. As discussed previously, these measurement models inherently consider the reliability and validity of observed metrics. Should our metrics have poor theoretical or empirical properties in this sense, erroneous conclusions regarding the diversity‐disease relationship are inevitable. Evidence for these causal relationships is critical in order to justify the continued interpretation and usage of these metrics in future studies and to inform the application of their findings in the realm of biodiversity conservation and public health initiatives (Kyle et al. 2020). Linking theory to practice is a common goal in disease ecology, and the explicit consideration of latent variables in the field is one step toward formalizing these concepts.

## 9 Conclusions

We advocate for a paradigm shift moving forward by placing less emphasis on net effect generalizations and by no longer testing the diversity‐disease relationship as a static, one effect size phenomenon occurring in a system. These traditional net effect approaches to the dilution and amplification effect framework are less helpful than they might have originally been in the past when exploring this emerging relationship between diversity change and zoonotic disease transmission in the literature. Since their proposal, these preliminary hypotheses have generated new, mechanistic hypotheses that we ought to lead with in future studies (Allan et al. 2010; Burkett‐Cadena et al. 2021; Figueroa et al. 2020; Gandy et al. 2021, 2022; Ginsberg et al. 2021; Kocher et al. 2023; Levi et al. 2012; Levine et al. 2016; Linske et al. 2018; MacDonald et al. 2022; Young et al. 2017). By shifting our focus to falsifiable mechanistic hypotheses and reframing our understanding of biodiversity and disease risk as latent variables, we believe that we can learn more about the diversity‐disease relationship.

## Author Contributions

**Shirley Chen:** conceptualization (equal), data curation (equal), formal analysis (equal), investigation (equal), writing – original draft (equal), writing – review and editing (equal). **S. Eryn McFarlane:** conceptualization (equal), supervision (equal), writing – review and editing (equal).

## Conflicts of Interest

The authors declare no conflicts of interest.

## Data Availability

All data and code for this project can be found at https://github.com/scjpg/lyme‐biodiversity‐biol4000.
